# Combinatorial (bio-geo-temporal) and non-combinatorial analysis of the COVID-19 dissemination that affected Georgia (the country) in 2021

**DOI:** 10.3389/fpubh.2026.1685435

**Published:** 2026-05-29

**Authors:** S. D. Smith, E. M. Geraghty, T. Goldstein, A. L. Rivas, F. O. Fasina, M. Kosoy, P. Imnadze, L. Malania, L. Kandelaki, I. Burjanadze, A. L. Hoogesteijn, T. C. Collins, R. K. Pilla, J. M. Fair

**Affiliations:** 1 Geospatial Research Services, Ithaca, NY, United States; 2 Esri, Redlands, CA, United States; 3 One Health Institute, Colorado State University, Fort Collins, CO, United States; 4 School of Medicine, University of New Mexico, Albuquerque, NM, United States; 5 Department of Veterinary Tropical Diseases, University of Pretoria, Onderstepoort, South Africa & Food and Agriculture Organization of the United Nations, Nairobi, Kenya; 6 KB One Health LLC, Fort Collins, CO, United States; 7 National Center for Disease Control & Public Health, Tbilisi, Georgia; 8 Department of Human Ecology, CINVESTAV, Merida, Yucatan, Mexico; 9 College of Population Health, University of New Mexico, Albuquerque, NM, United States; 10 University of Milan, Milan, Italy; 11 Biosecurity, Los Alamos National Laboratory, Los Alamos, NM, United States

**Keywords:** cost-effectiveness, COVID-19, epidemiology, Geographical Information Systems, Georgia (country)

## Abstract

**Introduction:**

To extract more information from the same data and support decision-making, we explored whether bio-geo-temporal (BGT) variables could distinguish municipalities where BGT variables differed and (if prioritized in subsequent interventions) could lead to cost-effective decision-making. This study was conducted investigating COVID-19 outbreaks reported in Georgia in 2021.

**Methods:**

Using both commercial and non-commercial proprietary software packages, we explored the test positivity rate or TP % (percentage of test-positive results among tested individuals), the date and the georeferenced location of municipalities where tests were conducted, as well as the associated population and road density (municipal road length / municipal area). Analyses included spatial (Getis-Ord and Moran’s I) and non-spatial statistical tests.

**Results:**

While the TP% was not linearly related with any one variable, combinations of BGT variables displayed distinct data patterns that separated two groups of municipalities (one composed of just two municipalities). The two-municipality group showed a statistically significantly greater median road density than the remaining municipalities. The same two municipalities also possessed a greater ability to detect asymptomatic cases: they expressed 20.3 times larger TP%/km^2^ but occupied a territory 18.3 times smaller than that of other municipalities. Combinatorial analyses that included testing emphasis (tests conducted per thousand inhabitants) provided novel ways to monitor and/or adjust testing policy at local levels. In contrast, analysis of the same adjusted TP% with three spatial statistical approaches identified much larger territories to be intervened.

**Discussion:**

Findings supported the view that methods exploring dynamic combinations of BGT relationships may identify highly connected municipalities (those likely to behave as network nodes during disease dissemination processes) when tested as TP%/km^2^. If selectively intervened, such nodes may lead to earlier cessation of epidemics. To explore the reproducibility of this proof-of-concept, further studies are recommended.

## 1 Introduction

The COVID-19 pandemic revealed complex relationships involving human behavior, viral transmission, the environment, and time, as well as major gaps in our ability to respond effectively, especially during the early stages of an outbreak (1). Key questions emerged during this pandemic: *where* and *when* should interventions occur, and *how* could silent spreaders (asymptomatic individuals) be identified before widespread transmission takes hold? These questions are particularly challenging because asymptomatic individuals often evade detection by traditional surveillance methods. Moreover, this disease is associated with a large percentage of asymptomatic people (2). Because asymptomatic but infected individuals spread the virus (3), controlling epidemics in their early stages is difficult when many cases are non-observable.

The first effort to develop a COVID 19-related testing policy took place in March of 2020, when World Health Organization (WHO) experts mentioned “test positivity” [TP or the percentage of diagnosis-positive tests when conducted at a certain time and place (4)]. Later studies found that the TP% is a good approximation of prevalence. If investigated in early epidemic stages with massive testing–, TP % is claimed to save lives (5–8).

Several epidemics have shown cyclic waves or phases (9). While reported in COVID-19, such TP-related waves have not yet been investigated with high-resolution (county-level) geo-referenced data (10–14). While attempts to relate TP to waves that express the relative relationship between susceptible and infected individuals have been made (15), such studies have lacked high-resolution, geo-refenced data and/or variables that reflect the testing-related policy.

Despite its early use as a public health metric, test positivity remains underexplored: when the keyword “COVID-19” was searched in the *Web of Science*^©^, 833,835 references were retrieved on Apr 18, 2025. However, when the phrase “test positivity” was added, only 3,992 references were found (0.005%). When, in addition, the prefix “geo” was included in the search, only 3 references were retrieved (0.000004% of the total).

This relative lack of research on the use of TP % matters because, in May of 2020, WHO recommended TP percentages <5 for at least 2 weeks before governments re-opened public activities previously under quarantines (16). Such recommendations appeared to suggest that TP at or below 5% would indicate the epidemic was under control.

Yet, when, at the end of 2020, the earliest studies on test positivity emerged, it was found that even 1% TP could be observed in protracted outbreaks (17). One year later, methods that captured the dynamic and complex properties of biological systems were still not available (18).

To fill some of these gaps, Systems Biology (SB) has been proposed. This method seeks to detect *unobserved data patterns* (19). To that end, *top-down* and *bottom-up* models are used.

Bottom-up models are based on correlation analysis. They assume that data are linearly and normally distributed. Such assumptions do not readily apply to dynamic biological systems, such as epidemics (20–22). In addition, bottom-up models tend to neglect complexity (23).

Top-down models may be classified as hypothesis-testing or hypothesis-generating (also known as data-driven) methods. While the former has been viewed as “confirmatory” and the latter as “descriptive” (or theory-free), such labels denote a dichotomy that does not always exist: data-driven research may also be generated by a theory (24).

Data-driven methods may generate inferences on rather limited units, such as a specific region inhabited by a specific sub-population at a specific temporal frame (25). Data-driven, methods are linked to pattern recognition, also known as data visualization analysis (26).

Data-driven, top-down research can benefit from prior knowledge and/or theory, such as Network Theory. Proximity to connecting networks –such as the road network– may predict disease dispersal (27–29). COVID-19 epidemics display Network Theory-related properties (30).

Because combinations of pre-existing geographical features influence epidemic dispersal (31), bio-geo-temporal (BGT) assessments might support epidemiologic interventions associated with lower costs and/or higher impact than those based on spatial statistics (32). The BGT analysis seems to capture relationships that reductionist approaches do not anticipate (33). Cost-effective, epidemiological methods can be viewed as combinatorial optimization approaches that may minimize the negative consequences caused by the spread of diseases (34).

Therefore, this study followed a combinatorial, data-driven, optimization-oriented approach that used data visualization technologies (e.g., Geographical Information Systems) and incorporated well-known theories, such as Network Theory (29, 35). By comparing a BGT method to analyses that utilize spatial statistical tests, the “nearest neighbor” theory was also tested (36). That is the theory that assumes neighbors located at short distances from one another (as defined by Euclidean distances) are more connected than distant ones and, consequently, in epidemics, “nearest neighbors” are expected to be predominantly affected (37, 38). Therefore, tests that investigate spatial auto-correlation (such as the Moran’s I and Getis-Ord tests) may detect “hot spots” or clusters of cases located within relatively small areas. However, the “nearest neighbors” theory and associated spatial statistical tests do not consider that disease dissemination may also result from combinations of variables that may follow pre-existing connecting networks –such as the road, railroad and/or river networks (27, 29).

## 2 Materials and methods

### 2.1 Data

The concepts identified above were investigated in Georgia (the country), where, in 2021, all weekly tests conducted in fifty-seven municipalities to detect COVID-19 were geo-referenced. The associated demographic data, the total number of diagnostic tests conducted per week and the weekly number of test-positive results were analyzed.

Testing-related, de-identified bio-geo-temporal data were provided by the National Center for Disease Control and Public Health, Tbilisi, Georgia and the Vakhushti Bagrationi Institute of Geography of Ivan Javakhishvili Tbilisi State University, Georgia. This study aimed to detect combined (BGT) interactions that could answer *where*, *when, why* and/or *how long* interventions that prioritized some geo-referenced targets (“epidemic nodes”) could be less costly and/or more beneficial. This study did not investigate interventions but the costs and/or benefits of BGT-centered interventions associated with testable theories on disease dispersal, such as the size of the area to be controlled (a “cost”, if larger than average), the number of cases identified (expected to be promptly isolated and treated; i.e., a “benefit”, if large), and a metric that expressed greater connectivity (such as a municipality with a higher road density than average). The unit of analysis was the municipality-specific, weekly Test Positivity percentage.

### 2.2 Methods

Using combinations of biological, geo-referenced and temporal (BGT) variables, we compared whether static, non-combinatorial and/or spatial statistical methods are as informative and cost-effective as combinatorial (BGT) ones. We regarded as “benefit” the proportion of cases potentially removed from the population when a context-specific, validated version of the TP% was used. To estimate “cost”, the territory covered by each method in the process of achieving the “benefit” was considered. For comparison, the same data were analyzed using the Getis-Ord GI* statistic (“hotspot analysis”) and the local Moran’s I tests (38). To generate combinations of BGT variables that elicit distinct (non-randomly distributed) data patterns that differ qualitatively across subsets (such as two or more subsets of municipalities that differed in some BGT variable[s]), a proprietary software package was used (U.S. Patent and Trade Office Provisional Application No. 63/608,670, 2023). This algorithm expedites the process required to combine and assess geo-referenced variables likely to be found or collected in any epidemic that occurs anywhere, including municipality-specific area and road density, population, time, the number of diagnostic tests conducted, the number of test-positive results obtained at a given time point at a specific site, and testing emphasis (tests conducted per thousand inhabitants). Specifically, this algorithm (a) generates data combinations (e.g., test positivity, road density, testing emphasis), (b) plots such combinations into 3D space, (c) applies unsupervised strategies that identify distinct patterns (e.g., orthogonal data inflections), and (d) based on such patterns, partitions the data into subsets. The process stops when non-overlapping data subsets emerge.

“Test positivity” (the percentage of test-positives) was calculated alone or combined with other variables. Combined indicators were identified with two- or three-letter identifiers in italics which lack BGT meaning. These indicators were no longer utilized after distinct patterns were detected. Then, visual patterns were used to partition the data into subsets subsequently explored with interpretable variables (e.g., the municipality-specific TP% found within a specific timeframe, which is associated with a specific road density, testing emphasis, etc.). Once the most informative metric was found, the size of its associated territory and the ability of the metric to promptly detect asymptomatic cases (as estimated by TP) was compared to solutions generated by spatial statistical tests. In addition, variables that may support or reject specific hypotheses were statistically compared with the Mann–Whitney test and regression analysis.

To validate this proof-of-concept, four threats were investigated, which addressed: (a) construct, (b) internal, (c) external and (d) statistical validity. Statistical validity is explored after the first three types are shown to be plausible. While external validity refers to reproducibility, internal validity addresses possible confounders that may induce artifacts or spurious results. Only when internal and external validity are supported, outcomes attributed to the central concept (the construct) become credible. To that end, two commercial software packages were utilized: *ArcGIS Pro 3.5* (Esri, Redlands, Ca) and *Minitab 22* (Minitab LLC, State College, Pa).

While interventions were not explicitly evaluated, proxy metrics assessed the ability of the method to generate cost-effective interventions. When BGT metrics suggested it could identify asymptomatic and symptomatic cases earlier and/or faster (because interventions would involve smaller areas with greater TP% than alternatives and such areas revealed greater connectivity than average), the proof-of-concept was deemed provisionally tenable.

## 3 Results

**Figure 1:**
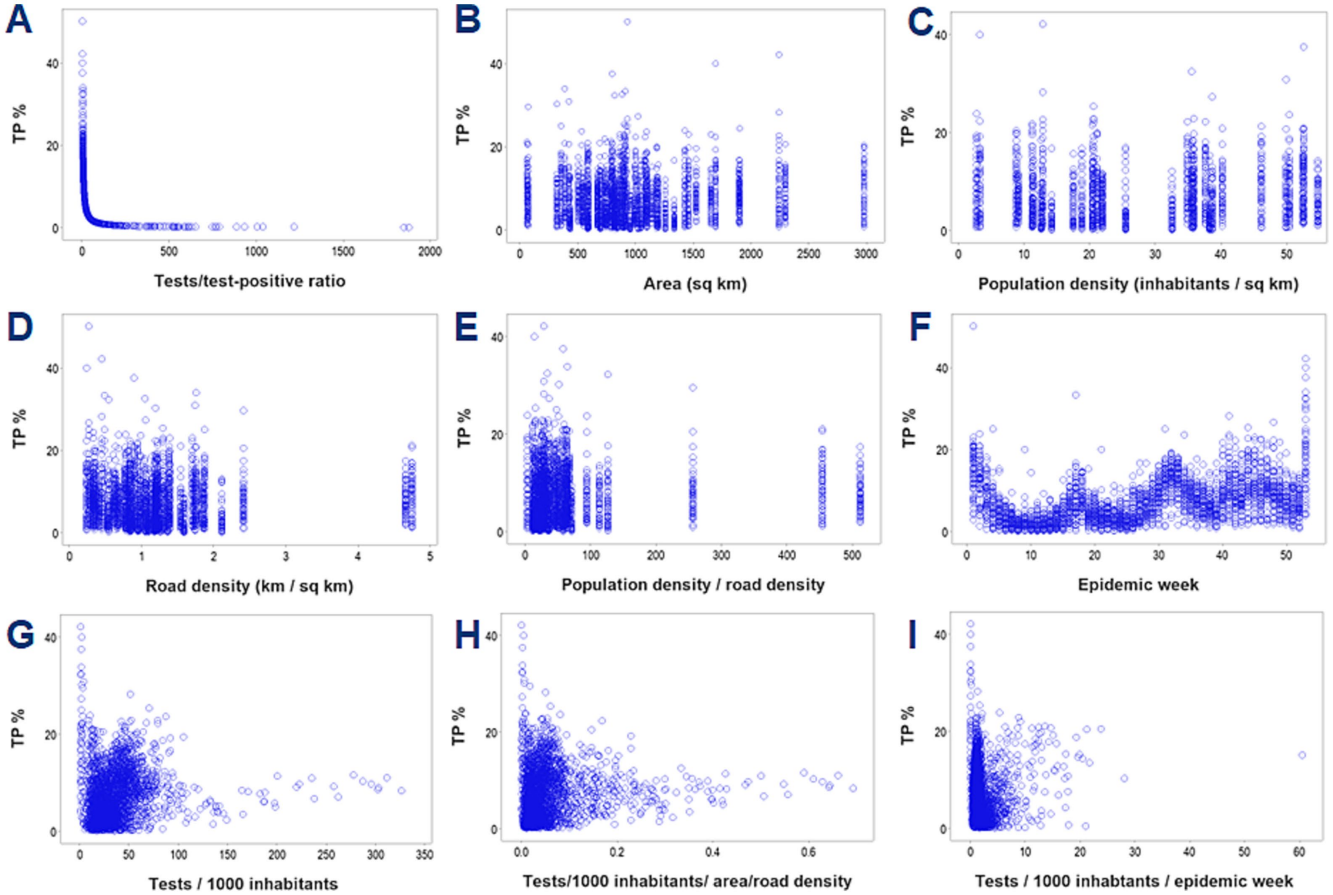
Descriptive analysis of individual variables. The percentage of non-adjusted test-positive tests (TP%) was not linearly correlated with any simple or combined variable under investigation **(A–I)**.

**Figure 2:**
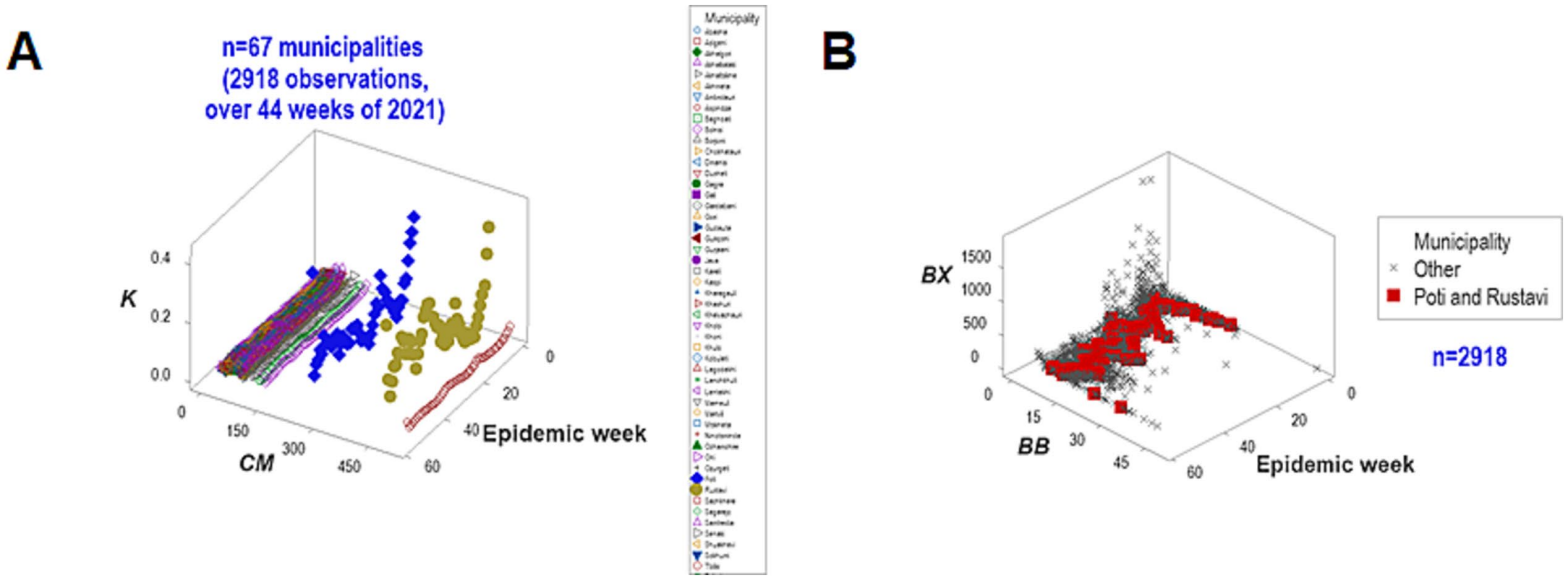
Emerging patterns generated by combinatorial indicators (I) where and why to intervene. Implemented by proprietary software, bio-geo-temporal (BGT) variables were integrated and analyzed as triplets and subsequently plotted in three-dimensional (3D) space. Synchronicity (a major feature of network theory) was demonstrated in two municipalities **(A)**. An overlapping pattern was observed when the two municipalities identified in (A) (Poti and Rustavi) were assessed together, which corroborated synchronicity even though these municipalities are located more than 300 km apart **(B)**.

**Figure 3:**
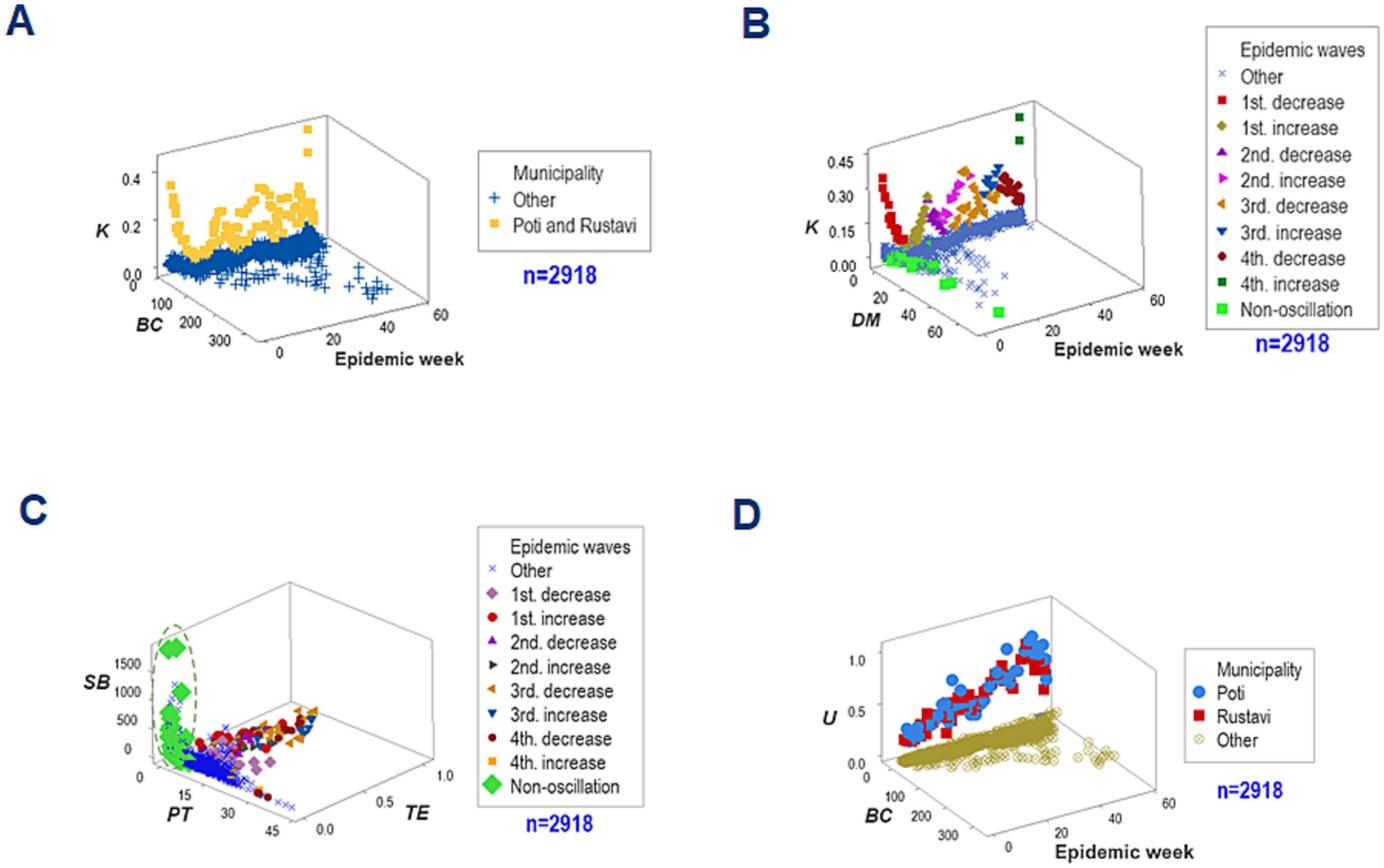
Emerging patterns generated by combinatorial indicators (II). When to intervene. When time was measured as epidemic weeks (weeks of 2021 when TP% was determined at municipality level), increasing and decreasing waves were observed in two municipalities (Poti and Rustavi, **A,B**). The simultaneous assessment of triplets of combinatorial indicators (which expressed at least seven interactions among primary variables) distinguished three data subsets perpendicular to one another **(C)**. Other data structures differentiated Poti and Rustavi from all other municipalities with non-overlapping data intervals **(D)**.

**Figure 4:**
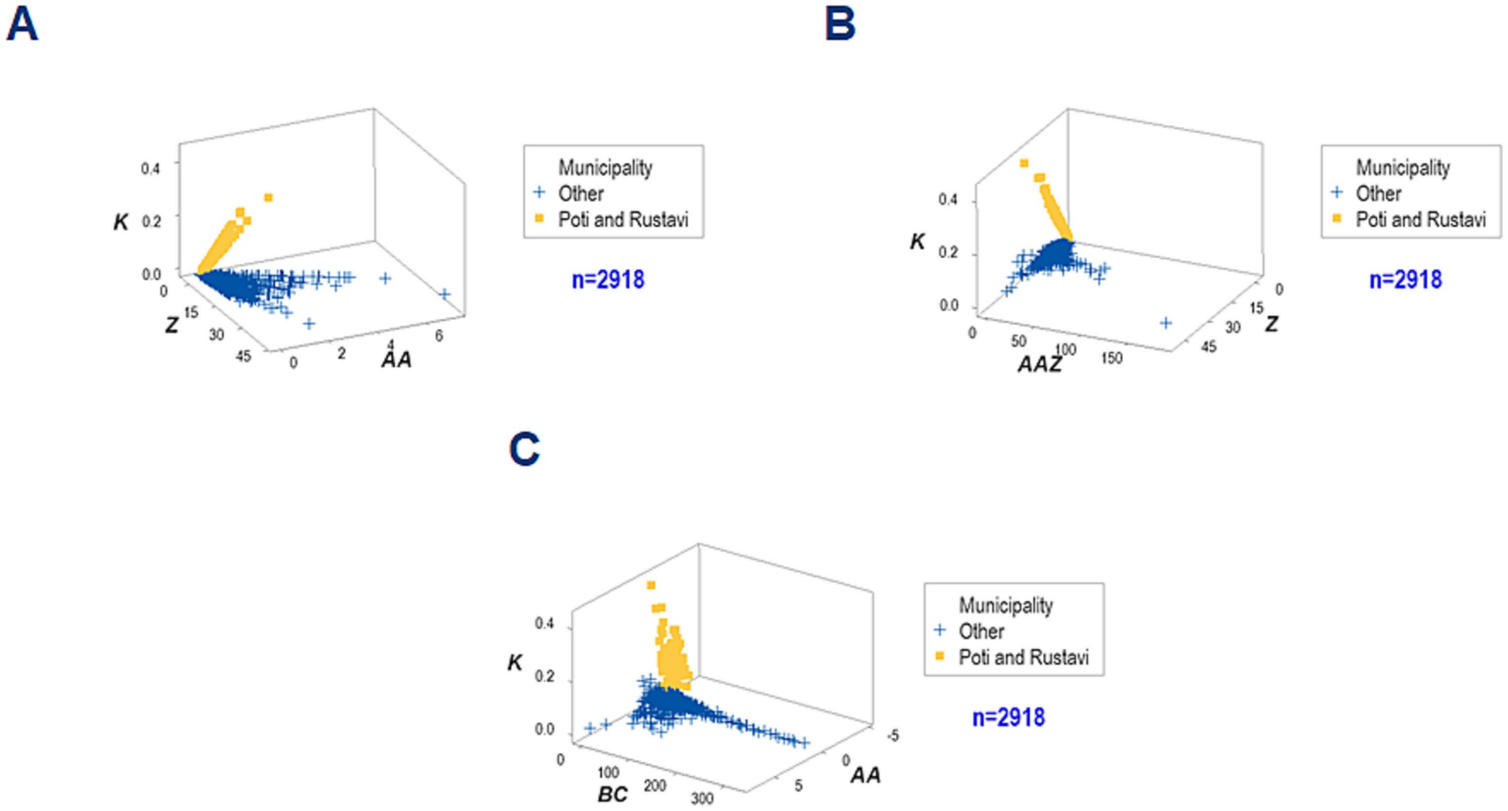
Emerging patterns generated by combinatorial indicators (III). Assessment of internal validity. Different data structures supported similar inferences **(A–C)**.

**Figure 5:**
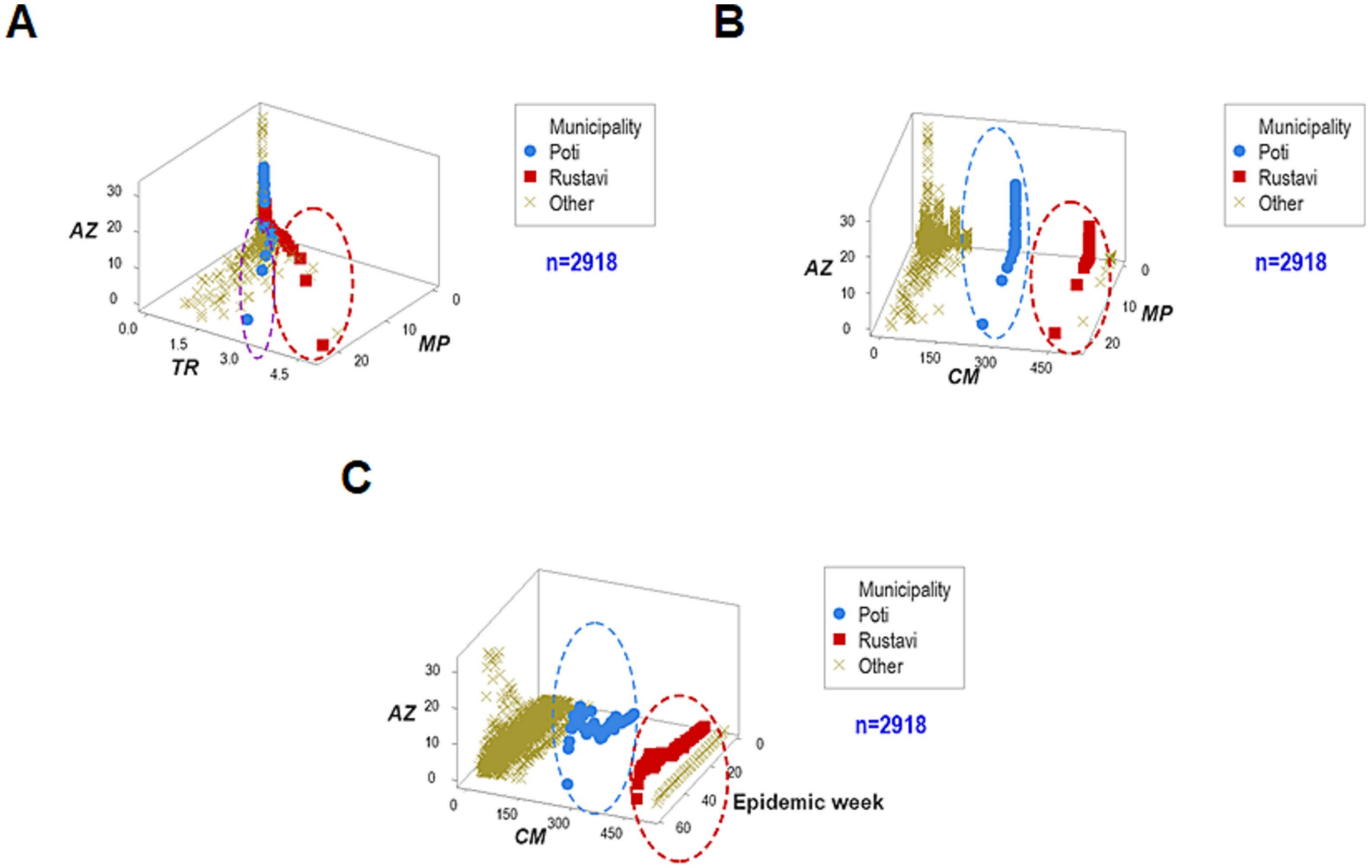
Emerging patterns generated by combinatorial indicators (IV). Discrimination within municipalities. In addition to differentiating two groups of municipalities, combinatorial analysis also distinguished Poti from Rustavi **(A–C)**.

The percentage of test-positives (TP %) was not linearly related with any one variable: no correlation explained more than 40% of the data variability (Figures 1A–I). Yet, several comparisons displayed patterns that suggested multi-dimensional relationships were informative. Distinct patterns emerged when three-dimensional (3D) plots that included two (or more) complex indicators that combined two or more variables were created (Figures 2–5).

*Where* and *why* to intervene and *what theory* was likely to explain the findings were explored with plots that revealed cyclic waves of observations. Such waves were synchronized: they occurred at the same time and revealed similar increasing or decreasing trends in two municipalities (Figures 2A,B). Because synchronicity is a major feature of network-related dissemination processes, findings supported the hypothesis that disease dissemination may be facilitated by network nodes (highly connected sites), which Poti and Rustavi appeared to be.

*Where* and *when* to intervene was investigated in four additional combinatorial analyses. When time was measured (expressed as epidemic week in reference to 2021), perpendicular relations differentiated Poti and Rustavi from the remaining municipalities (Figures 3A,B). Poti and Rustavi predominantly revealed wave-like changes over time, which were perpendicular to two other sets of municipalities (one revealing, the other not revealing data oscillations over time, Figures 3B,C). Such geometric patterns suggested that the differences between municipalities (characterized as either non-cyclic data points or cyclic waves) were not random events. An additional data structure revealed non-overlapping data patterns that unambiguously differentiated Poti and Rustavi from the remaining municipalities (Figure 3D).

The internal validity of this study was supported: similar inferences were found even when different variables were measured (Figures 4A–C). The BGT method also distinguished Poti from Rustavi (Figures 5A–C).

**Figure 6:**
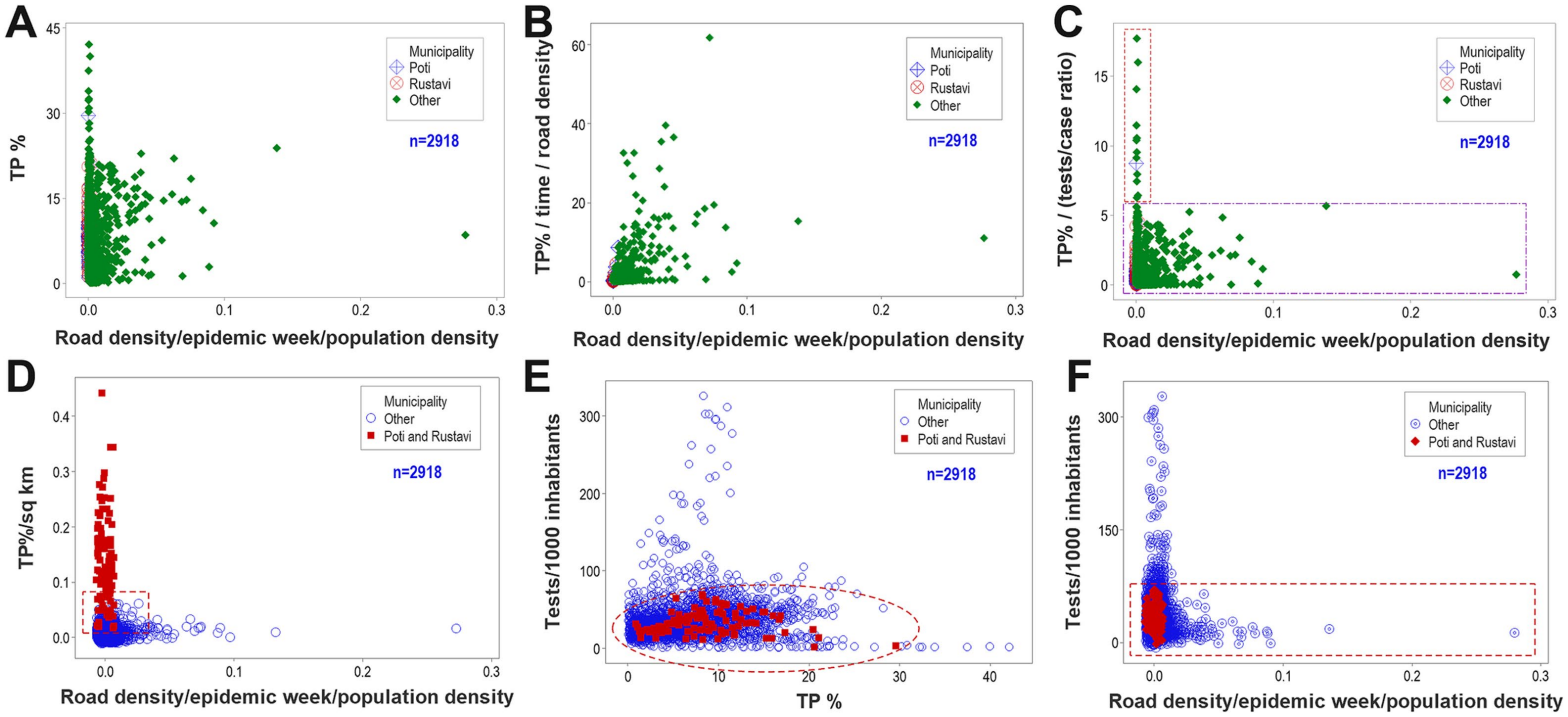
Non-informative combinations. Not all data combinations were informative even when a variable already known to be informative (the TP%) was included in the assessment **(A)**. Even when the TP% was combined with other variables, a substantial overlapping was observed **(B–D)**. Data overlapping remained even when the policy-related variable (testing emphasis or tests/1000 inhabitants) was considered (red rectangle or oval, **E,F**).

**Figure 7:**
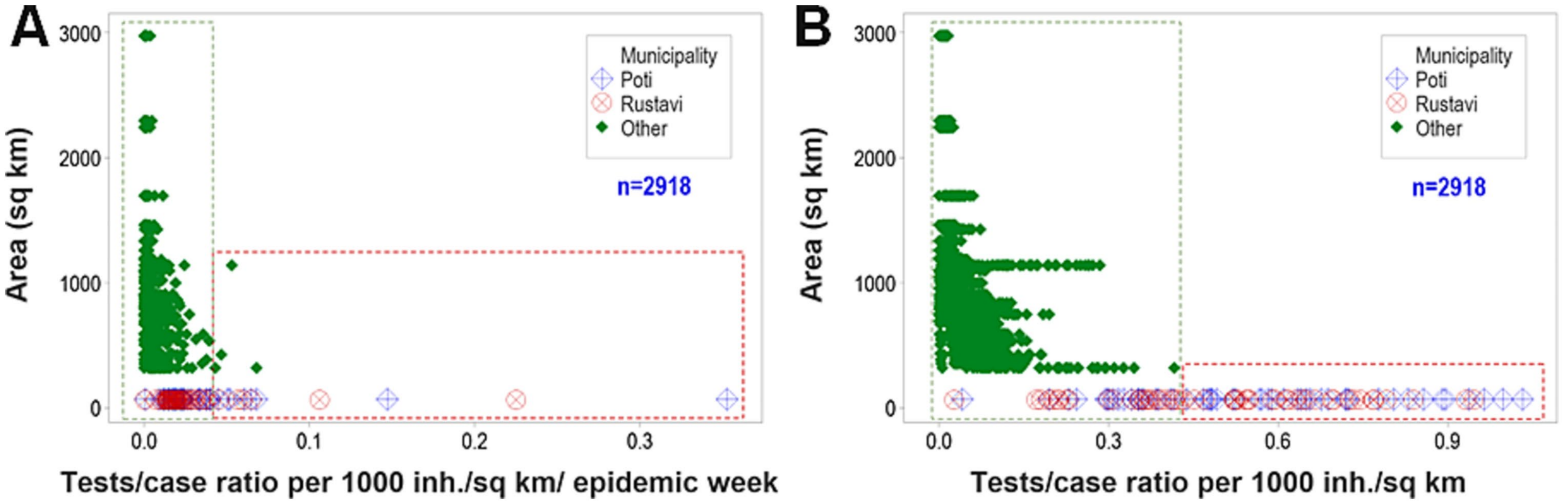
Alone, no variable was informative (II). Neither area nor time informed. Municipal area did not explain why Poti and Rustavi differed from the remaining municipalities **(A,B)**. Because epidemic weeks were not assessed when two Poti and Rustavi subsets were differentiated, it was demonstrated that time was not a critical differentiator, either **(B)**.

Yet, no variable, alone (including TP%), was informative (Figures 6, 7). For example, the non-adjusted TP percentage did not distinguish Poti and Rustavi from the remaining sites (Figure 6A). When combined with other variables, the TP% seemed to reveal some patterns but overlapping remained (Figures 6B–F). Similarly, the policy-related variable (testing emphasis), alone, did not differ significantly between Poti and Rustavi and the remaining municipalities regardless of the inclusion or exclusion of the TP%/km^2^ (Figures 6E,F).

Area and time did not inform, either. Poti and Rustavi differed from other sites not because of any one variable but due to complex interactions among variables (Figures 7A,B).

To investigate the factors that distinguished the two apparent epidemic nodes (Poti and Rustavi) from other municipalities, the area-adjusted, test positivity rate (TP%/km^2^) was analyzed with a regression that included road density (km/km^2^), population density (inhabitants/km^2^), and testing emphasis (tests per 1,000 inhabitants). The weight of Poti and Rustavi was ~148 times larger than those of the remaining municipalities (Supplementary Table 1). Because no evidence of valid and significant correlations among predictors was found (Supplementary Figures 1A–C), threats to internal validity were ruled out while construct validity (the hypothesis that TP%/km^2^ may be predicted by combined data structures) was supported.

**Figure 8:**
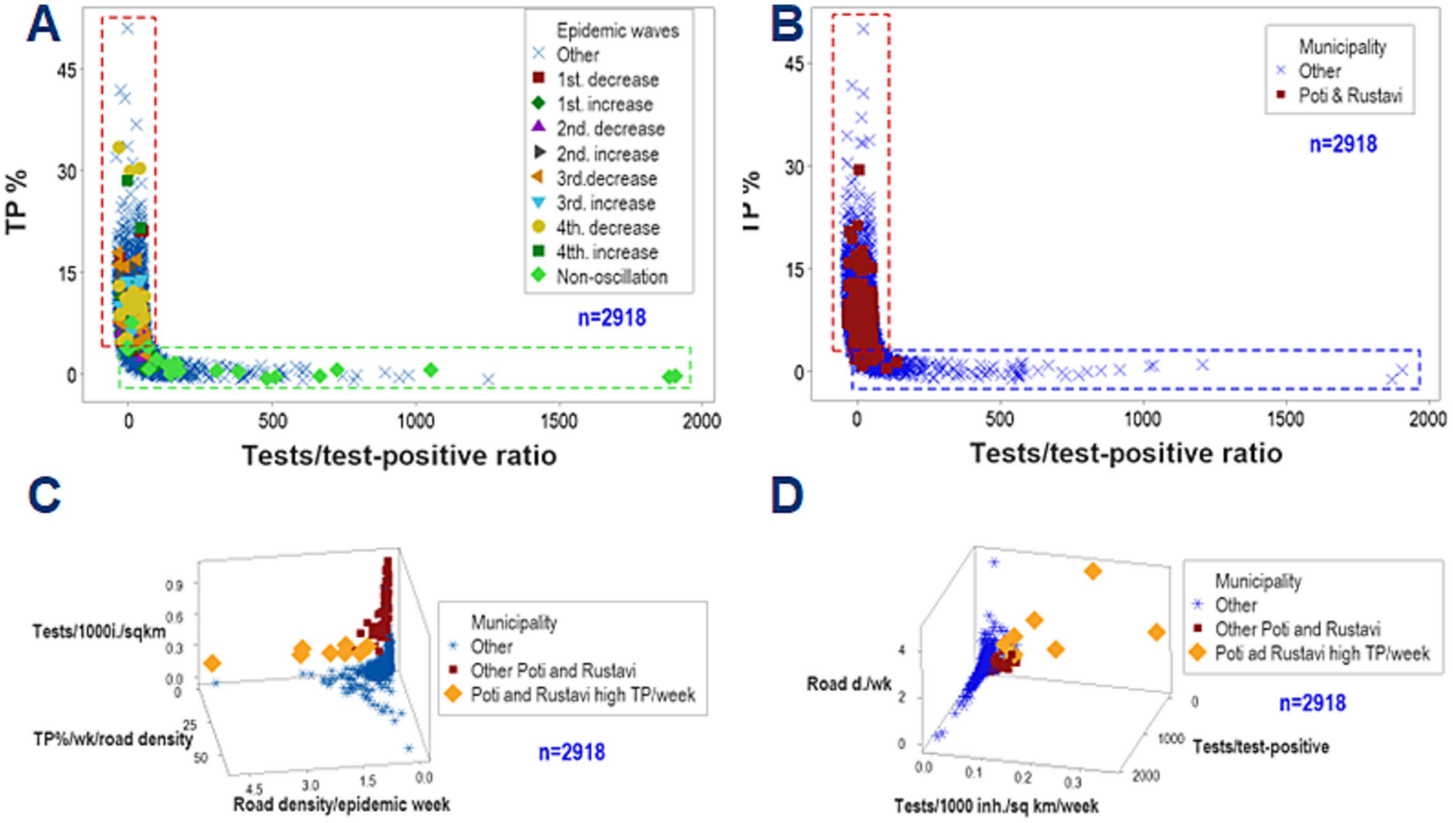
Additional non-linear relationships. The non-linear relationship between TP% and its inverse **(A,B)** indicated that BGT interactions are not likely to be validly captured by reductionistic methods. In contrast, non-reductionist (three-dimensional) assessments of triplets differentiated two of more data subsets **(C,D)**.

Other 2D and 3D analyses differentiated *betwee*n and *within* municipalities. For example, the inverse of TP% did not reveal a linear and negative relationship with TP% but an orthogonal one (Figure 8A). Such a pattern was explained by differences *between* municipalities: while Poti and Rustavi included most of the increasing and decreasing TP% oscillations (which represented the highest values of the TP%), the remaining values (which occupied the horizontal segment of the TP%) were contributed by other (“non-oscillating”) municipalities (Figure 8B).

**Figure 9:**
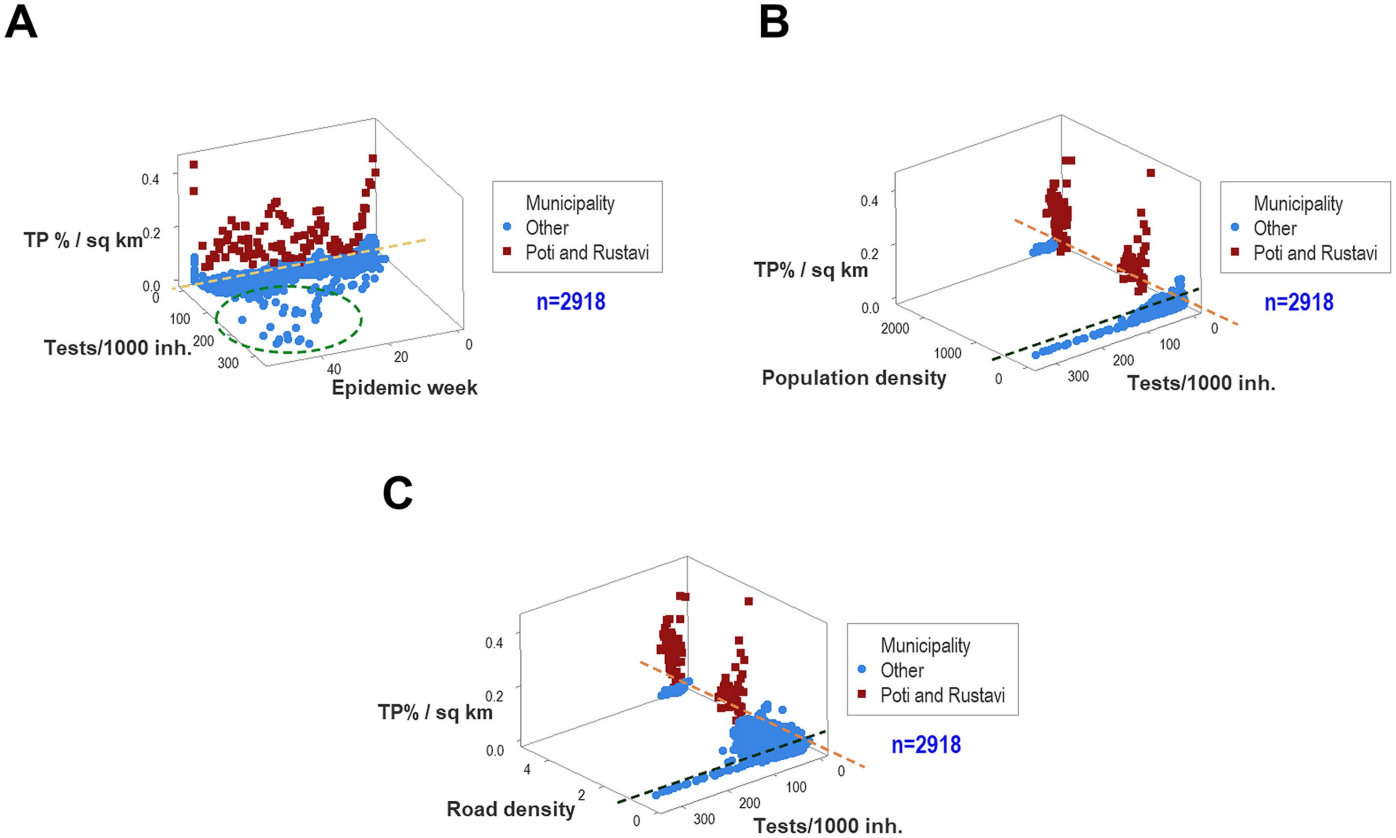
Non-overlapping, three-dimensional, and policy-related patterns. Non-overlapping information was only conveyed when the combinatorial approach considered 3D interactions **(A–C)**. For example, policy (measured as testing emphasis) differed across municipalities: it was up to 30% (300 tests/1,000 inhabitants) in several municipalities but less than 7% (70 tests/1000 inhabitants) in Poti and Rustavi (yellow horizontal line, **A**). Testing emphasis remained constant throughout 2021 in Poti and Rustavi but it increased, over time, in some municipalities (blue oval, **A**). On average, Poti and Rustavi displayed the highest road density, the highest population density, the highest TP, and the lowest testing emphasis **(B,C)**.

Data overlapping was only prevented when the combinatorial and dynamic nature of the BGT three-dimensional method was tested. Such analyses facilitated explanatory information that distinguished municipalities, providing real-time assessments that might indicate whether to start, stop, and/or adjust a specific policy (Figures 8C,D). For example, 3D plots showed different testing emphasis across sites and time: testing was up to 30% (300 tests/1000 inhabitants) greater in several municipalities but less than 7% (70 tests/1000 inhabitants) in Poti and Rustavi (yellow horizontal line, Figure 9A). Testing emphasis remained constant throughout 2021 in Poti and Rustavi, while it increased, over time, in some of the remaining municipalities (blue oval, Figure 9A). Poti and Rustavi also displayed high values in variables that relate to Network Theory, such as road density and population density (Figures 9B,C).

**Figure 10:**
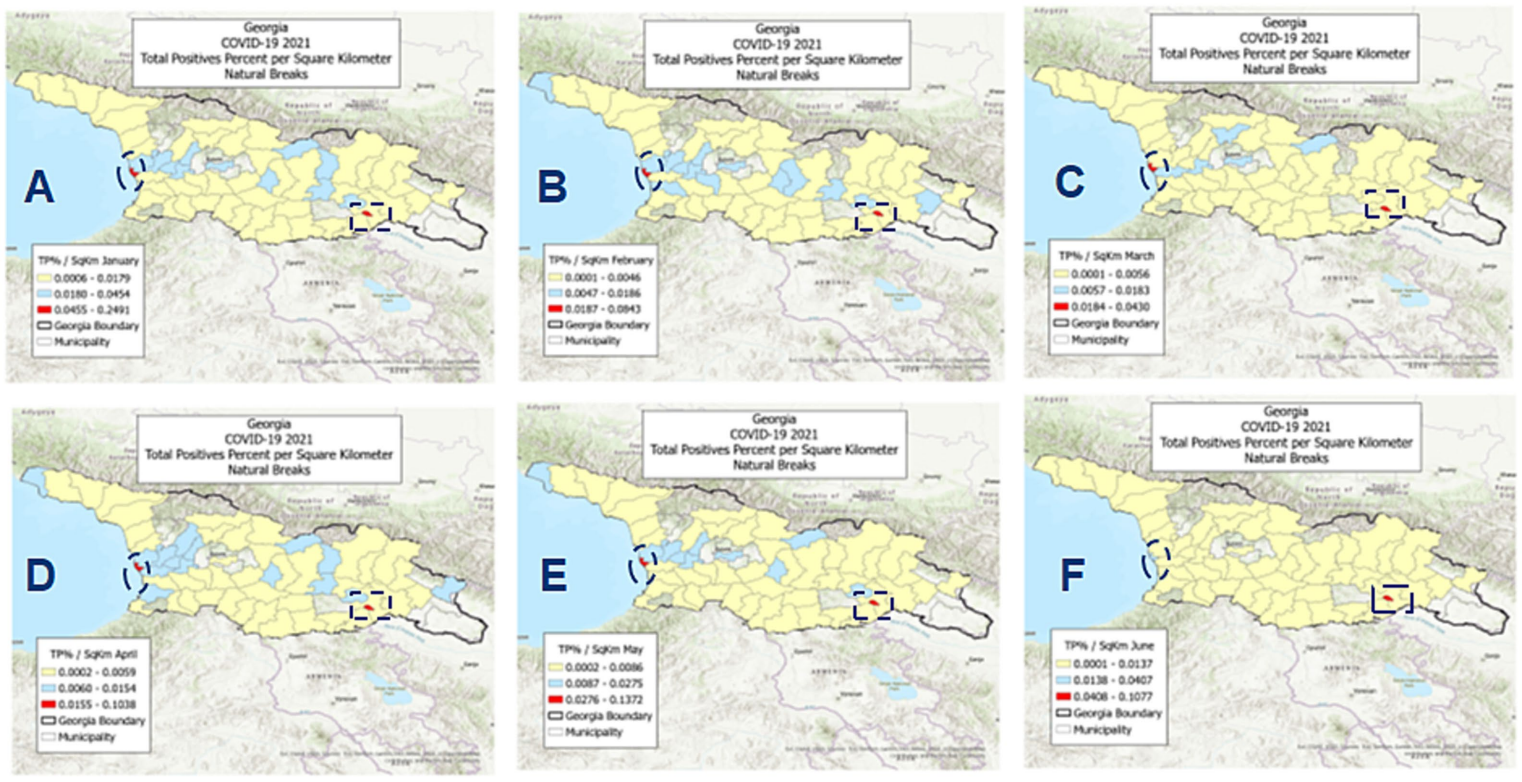
Geographical validation (I). Temporal consistency and real-time evaluations. External validity (demonstration of similar findings over time) was documented: over a six-month long period, Poti and Rustavi municipalities showed the highest TP%/ km^2^ in eleven out of twelve assessments **(A–F)**.

**Figure 11:**
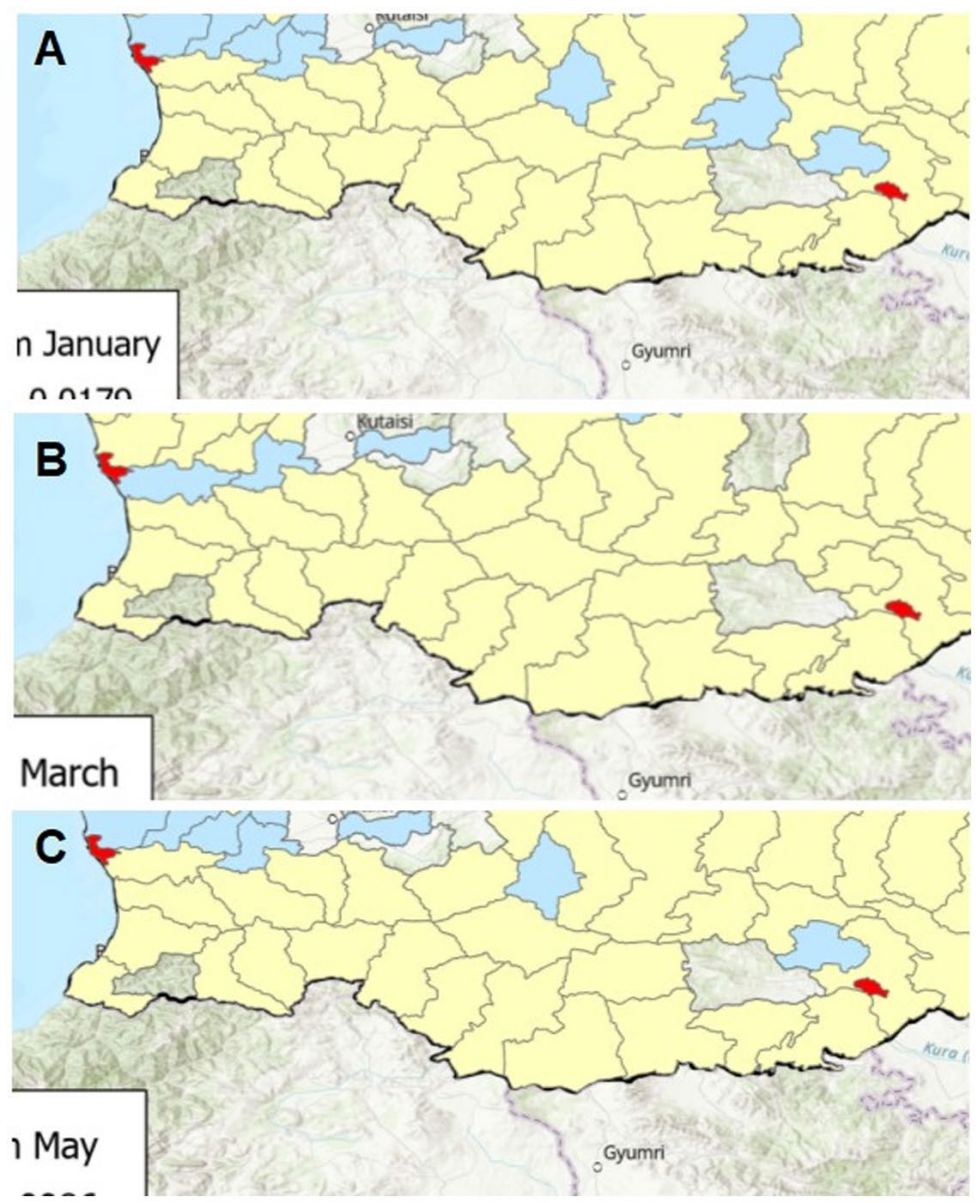
Geographical validation (II). Corroboration of earlier findings. A partial magnification of Figures 10A–F both supported findings that indicated disease dissemination is facilitated by connecting networks and disproved the “nearest neighbor” theory: the two municipalities that consistently showed the highest TP%/km^2^ were located 320 km apart (**A–C**: January, March, May of 2021).

**Figure 12:**
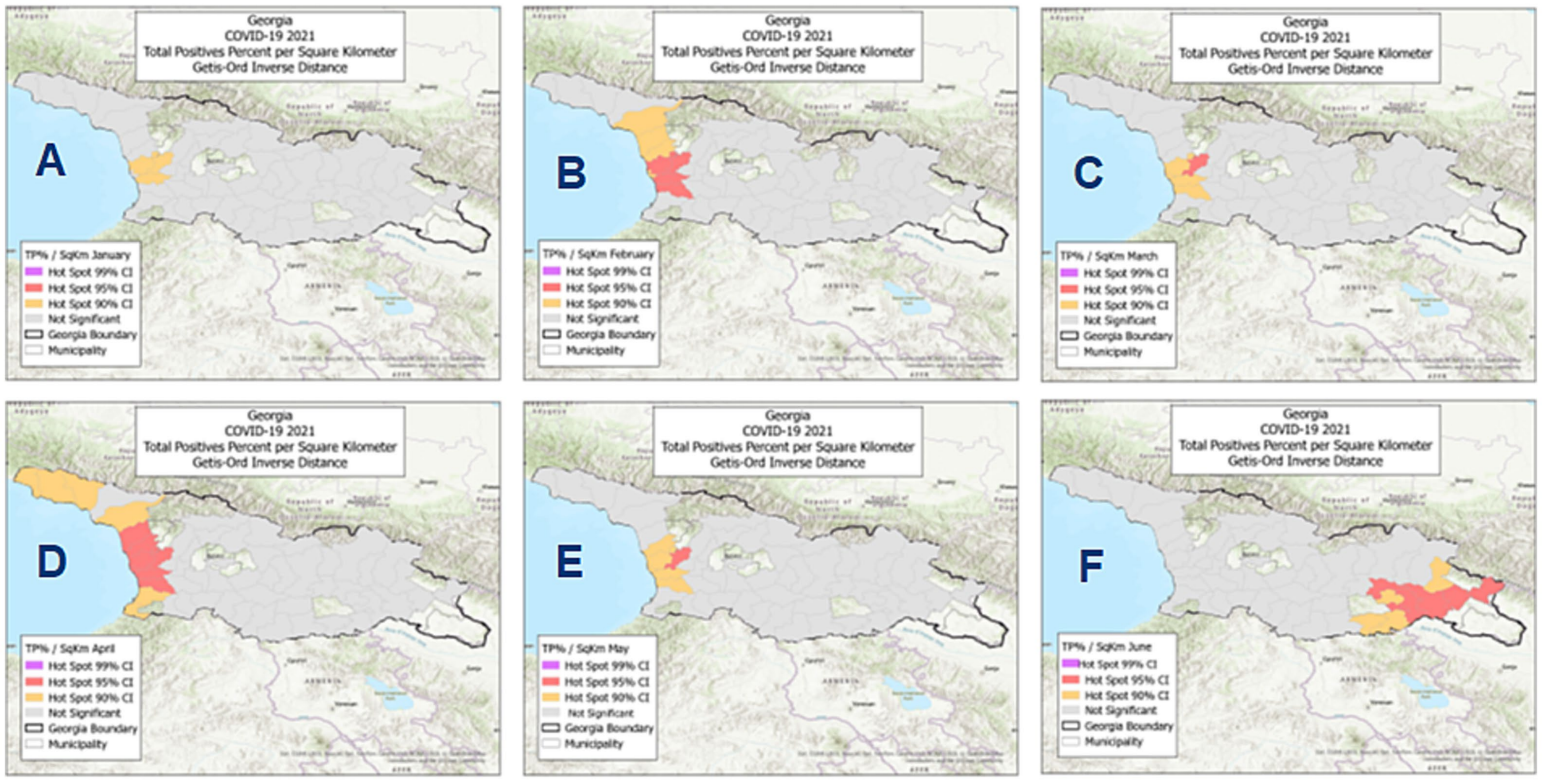
Getis-Ord test. Focusing on TP%/km^2^, the areas described by 90% or greater confidence intervals as calculated by the inverse distance method identify municipalities regarded as “hotspots” between January and June, 2021 **(A–F)**.

**Figure 13:**
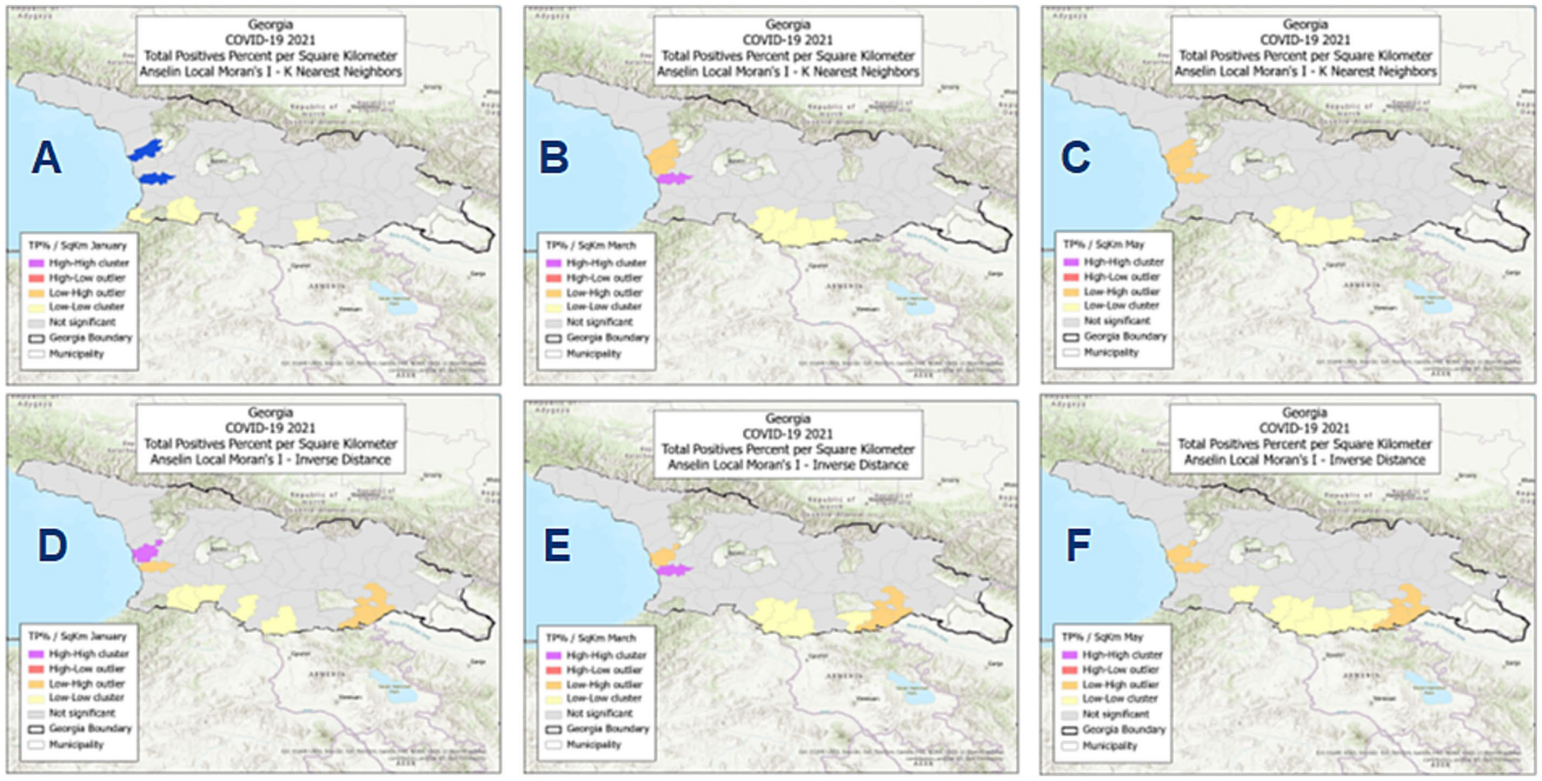
Local Moran’s I. Focusing on TP%/km^2^, two versions of the local Moran’s I test [the *K* nearest neighbor method, calculated for January, March, June 2021 **(A–C)**] and the inverse distance method, also calculated for the same months **(D–F)** identify clusters of auto-correlated areas.

The area-adjusted TP% showed temporal consistency. Over a year, Poti and Rustavi displayed the highest values in eleven out of twelve assessments (Figures 10, 11). In contrast, all spatial statistical analyses displayed temporal inconsistency: different sites were captured in every pair of consecutive measurements and all of them involved larger areas than the BGT alternative (Figures 12, 13). Geo-temporal data did not support the hypothesis that Poti and Rustavi were “nearest neighbors”: they are located >320 km from one another (Figures 11A–C).

**Figure 14:**
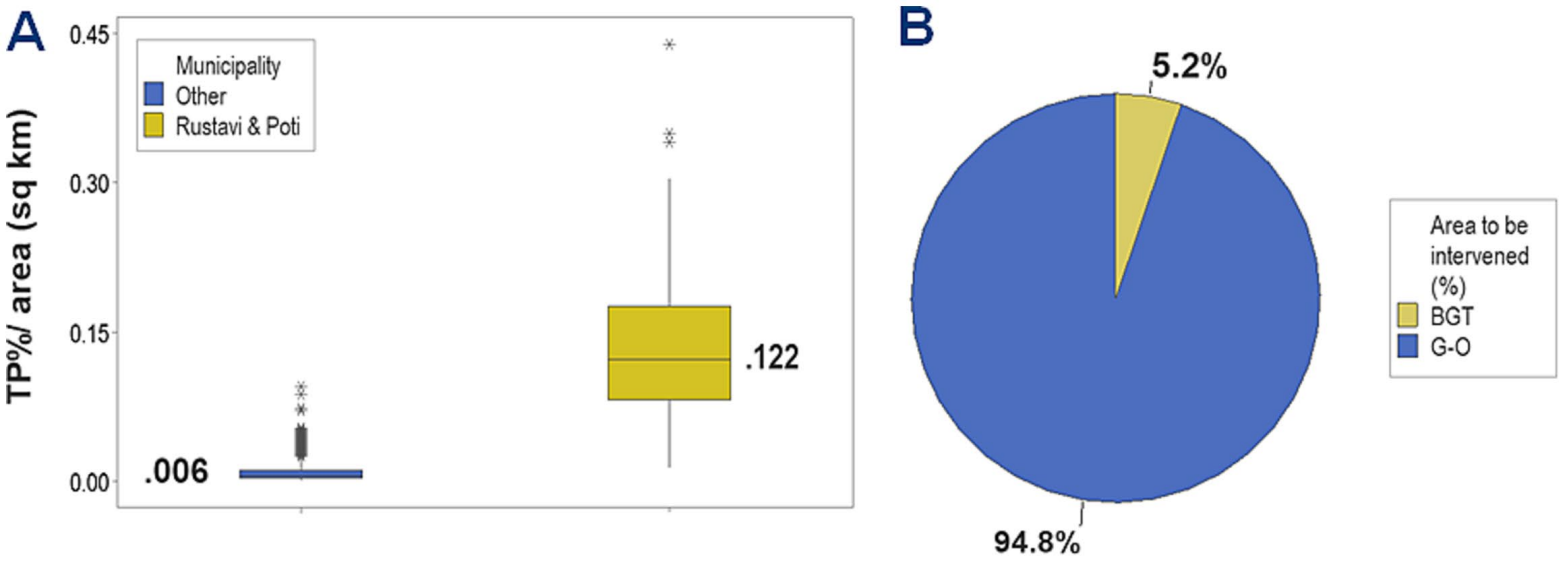
Cost-effective comparison between the BGT and the spatial statistical solution. The TP%/km^2^ was 20.3 times higher in the area identified by the BGT method than the one associated with the Getis-Ord (GO) solution [0.122/0.006, **(A)**]. The GO-based intervention would require covering an area 18.2 times larger than the one associated with the BGT approach [94.8/5.2, **(B)**].

**Figure 15:**
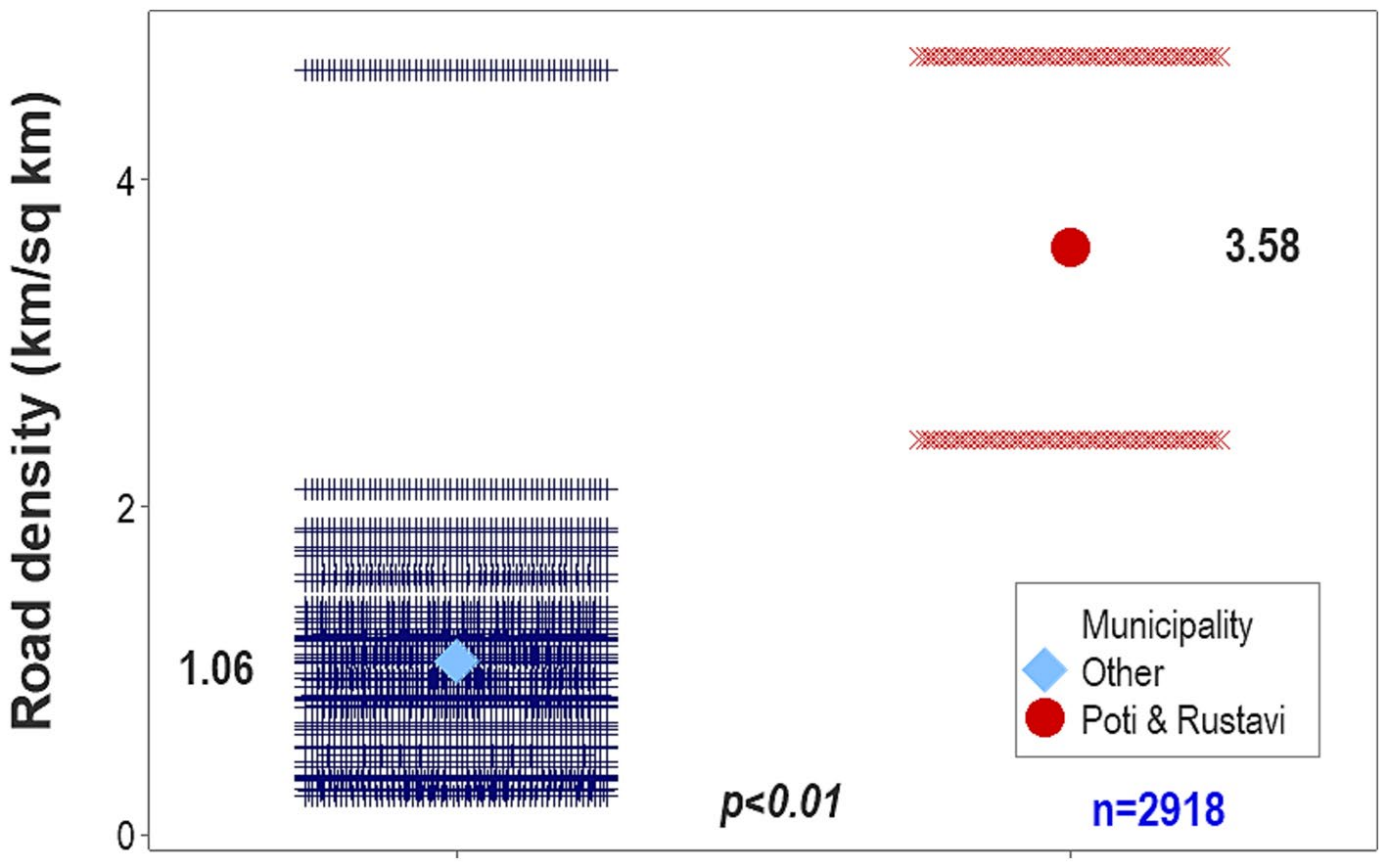
Network theory-related justification and statistical validity. Both geographically explicit dissemination of the epidemic through highly connecting network nodes and the statistical validity of the BGT method were supported when a combined variable of relevance in networks was measured (the road density or length of roads [km] adjusted to municipality-specific area [km^2^]). It showed the two municipalities suspected of acting as major network nodes displayed a ~ 3.5 higher median road density than the remaining municipalities (*p* < 0.01, Mann–Whitney).

The BGT method could, potentially, identify asymptomatic and symptomatic cases 20.3 times faster or earlier than the solution offered by the Getis-Ord test (TP %/km^2^ were 0.122 and 0.006, respectively, Figure 14A). In addition, the BGT method would require a 18.2 times smaller area than the policy based on the Getis-Ord test (94.8/5.2, Figure 14B). Thus, a BGT-based policy could detect (and consequently, treat and isolate) cases faster than the alternative. Statistical validity was also documented: the median road density was ~3.5 times greater in Poti and Rustavi than in the remaining municipalities (*p* < 0.01, Mann–Whitney, Figure 15).

## 4 Discussion

### 4.1 Overview

This study explored an epidemiological, data-driven, combinatorial method that focuses on pattern-recognition. Because the core variables used in this study – location, time, population, testing data, and infrastructure – are typically available in most public health surveillance systems, the BGT approach may have broad potential applicability. However, the specific relationships and data patterns identified here are context-dependent. Prospective studies in other geographies and disease contexts are needed to further validate and/or adapt this methodology.

A major finding was that discriminant information was not based on any one variable measured in isolation but combinations that included an adjusted version of Test Positivity (TP%/km^2^). Another principal finding was that data combinations that included the adjusted TP facilitated a greater and faster identification of asymptomatic cases than methods based on the “nearest neighbor” theory. A novel finding was the detection of cyclic waves of increasing and decreasing TP levels, which were predominantly observed in two municipalities. Because the BGT method also informed on where, why, when, how much and/or how long a specific policy could start, stop or change, and because such features were consistent with concepts broadly validated in epidemiology (Network Theory), this proof-of-concept was provisionally viewed as tenable and deemed to be applicable when case identification is problematic.

### 4.2 Differences between assumption-free and assumption-driven methods

We compared a flexible, data-driven combinatorial approach with spatial statistical methods, such as those based on the “nearest neighbors” theory (when defined according to relatively short Euclidean distances). Analytical tools that measure geographical contexts have shown many exceptions to the “nearest neighbor” theory, e.g., a railroad can rapidly connect neighbors that seemed to be distant. This study corroborated earlier epidemiologic studies on the higher connectivity captured by geo-referenced epidemic networks (as estimated by road density) compared to alternatives based on “nearest neighbors” analysis (27, 29).

Yet, findings should not be construed as opposing the use of spatial statistical methods. While spatial statistics are not intended to capture long-distance, connecting and dynamic networks [unless such networks are assessed considering “along-the-road” (non-Euclidean) models (37–39)], context and research needs may justify the use of such methods.

### 4.3 Applications in cost-effective decision-making

While this study did not evaluate implemented policies, it estimated the potential “cost” and “benefit” of a hypothetical policy meant to identify asymptomatic cases earlier. A higher benefit achieved at a lower cost was viewed as the smallest area required to cover the highest TP%/km^2^ –a metric estimated to indicate a high percentage of asymptomatic cases. The BGT method also provided context-specific (municipality- and time-specific), information on how to adjust, start or stop a policy. After the combinatorial algorithm differentiated two municipalities from the remaining ones, it was possible to revise, continuously, the relationships between testing emphasis and time (Figure 9B). This means that, instead of a fixed (constant) testing emphasis conducted homogeneously over a country over time (which may be insufficient to remove asymptomatic cases and, consequently, could lead to epidemics affecting more people over longer periods), a BGT-centered policy that emphasizes testing in small and/or highly connected areas exhibiting higher TP% may be more effective (17, 40, 41).

Thus, the BGT method may integrate scientific, ethical, and managerial challenges into a single (although flexible) operation. As unfortunately experienced many times in 2020, an exponentially growing epidemic can rapidly deplete critical resources (such as hospital beds, oxygen, and ventilators). Because exponential problems are easily explained but, usually, poorly assimilated (42), this methodology is aligned with a strategic managerial need: how to prioritize interventions that deliver the greatest effect, earlier, and prevent later detrimental outcomes, such as “long” COVID-19 and infections erroneously thought to be eradicated (43, 44).

### 4.4 Prevention of errors

*Ecological fallacies* are errors that occur when predictions are made about individuals assumed to behave identically –a problem observed when inferences are based on data aggregates. Because such aggregates merge all data collected from a given population, they do not capture internal relationships (45, 46). The BGT method prevented such errors as well as those that assume geographical entities are internally homogeneous (47).

Static methods tend to miss information on dynamics –e.g., the several phases of local outbreaks, which are not captured by spatial statistics (31, 35). Such methodological differences may explain why municipality-specific cyclic waves had not been reported before in small areas.

### 4.5 From a static focus on isolated variables to combinatorial and dynamic interactions

The BGT method was not reductionistic: discrimination was not due to a single and/or static variable measured in a single (biological, geographic, or temporal) domain. Instead, numerous fields were explored, such as epidemiology, diagnostics, economics, and decision-making (48, 49). While reductionist methods investigate isolated *entities* (i.e., variables), the combinatorial and dynamic BGT approach measures *relationships* among entities (50).

To prevent erroneous inferences, the contribution of Poti, Rustavi and other municipalities, as well as road density, population density and testing emphasis was assessed in a multiple linear regression that measured the adjusted TP%/km^2^ as the outcome. Supplementary Table 1 reports a multiple linear regression in which the weight of Poti and Rustavi was ~148 times larger than those of the remaining municipalities and predictors. Furthermore, road density, population density, and testing emphasis were not correlated validly and significantly because: (a) the correlation between road density and population was an artifact (at least two outliers were observed and the generated line was a curve, not a straight line, Supplementary Figure 1A) and (b) none of the other correlations showed linearity (Supplementary Figures 1B,C).

Yet, correlation analysis, alone, is not well fit to capture the changing (dynamic) and quasi-exponential growth of epidemics. For example, the correlation between population density and (COVDI-19 related) fatalities was not significant when tested in all 50 states of the US one month after the first case was reported but it was statistically significant three months later (51). The likely reason for such a difference is that, in the first assessment, the epidemic had not yet become fully consolidated (it was not yet disseminating along one or more connecting network[s]) while, three months later, connecting networks facilitated epidemic growth.

Furthermore, qualitative information was also considered. Both Poti (the only international port of Georgia) and Rustavi (a railroad and road hub, close to Georgia’s only international airport) show empirical evidence that supports their role as network nodes (52).

### 4.6 Methodological considerations and testing policy

Combinatorial methods have a long history in Biology but a brief one in Epidemiology. While a journal on combinatorial chemistry emerged in 1999 and combinatorial immunology already has some experiences (53–56), combinatorial epidemiology only recently started (31).

One potential focus of this new field is optimization of solutions for problems related to feedback and multiple interactions (57). Combined with machine learning, BGT studies could seek optimization without prior knowledge on the best combination (58).

### 4.7 Educational implications

The hypothesis that *high TP percentages reflect a substantial number of undetected cases* is supported by the fact that, in 2020, 51 countries with test positivity > 5% reported, on average, 15 times more deaths than countries that exhibited <1% test positivity (17). Since April of 2020, testing policies that foster the prompt removal of asymptomatic cases have been proposed (59).

Barring a few exceptions that focus on testing wastewater (60), the recommendation mentioned above has not yet been adopted. To fill that gap, new educational programs are needed. They could include skills that help integrate knowledge in policy-making (61).

### 4.8 Limitations of this study

While spatial statistical alternatives were explored, many other possibilities (e.g., the Geographically Weighted Regression) remain to be considered (39). Future studies could also look for new (a) estimates of “cost” and “benefit”, (b) mechanisms that foster rapid deployment of critical resources, and (c) methods that link BGT perspectives with Network Theory. Because no retrospective study can rule out all threats to validity, prospective studies that use biomedically interpretable benchmarks –such as serology– are needed to support or reject these findings (43).

## 5 Conclusion

Findings emphasize the role of pattern recognition when combinations of bio-geo-temporal variables are investigated –not any one numerical value or variable, measured alone and/or at a single time point. Given the inter−/trans-disciplinary nature of a combinatorial methodology, novel educational programs that integrate knowledge across fields and foster policy-making are recommended. If corroborated, this method may offer new alternatives to improve outbreak response, optimize testing policies, and make better informed decisions, even under conditions of uncertainty and substantial numbers of asymptomatic individuals.

## Data Availability

The original contributions presented in the study are included in the article/Supplementary material, further inquiries can be directed to the corresponding author.
