# Supplementary material for: Combinatorial (bio-geo-temporal) and non-combinatorial analysis of the COVID-19 dissemination that affected Georgia (the country) in 2021
Source: Front Public Health. 2026 May 29;14:1685435. doi: 10.3389/fpubh.2026.1685435 (PMC13260472; doi:10.3389/fpubh.2026.1685435)
Supplement: Supplementary file 1 [file Supplementary_file_1.docx]

**Supplementary material**

**Combinatorial (bio-geo-temporal) and non-combinatorial analysis of the COVID-19 dissemination that affected Georgia (the country) in 2021**

Smith SD^1^, Geraghty EM^2^, Goldstein T^3^, Rivas AL^4*^, Fasina FO^5^, Kosoy M^6^, Malania L^7^ Kandelaki L^7^, I. Burjanadze I^7^, Hoogesteijn AL^8^, Collins TC^9^, Pilla RK^10^, Fair JM^11^

^1^ Geospatial Research Services, Ithaca, NY, United States

^2^Esri, Redlands, California, United States

^3^One Health Institute, Colorado State University, Fort Collins, CO, United States

^4^ School of Medicine, University of New Mexico, Albuquerque, NM, United States

^5^ Department of Veterinary Tropical Diseases, University of Pretoria, Onderstepoort, South

Africa & Food and Agriculture Organization of the United Nations, Nairobi, Kenya.

^6^KB One Health LLC, Fort Collins, CO, United States

^7^National Center for Disease Control & Public Health, Tbilisi, Georgia

^8^Department of Human Ecology, CINVESTAV, Merida, Yucatan, Mexico

^9^College of Population Health, University of New Mexico, Albuquerque, NM, United States

^10^ University of Milan, Milan, Italy

^11^Biosecurity, Los Alamos National Laboratory, Los Alamos, NM, United States

***Address correspondence:** Dr. Ariel L. Rivas., e-mail: [alrivas@unm.edu](mailto:alrivas@unm.edu)

**Supplementary Table 1:** [**https://drive.proton.me/urls/XGZ5ZGEKV0#nRgVGeyHtu4V**](https://drive.proton.me/urls/XGZ5ZGEKV0#nRgVGeyHtu4V)

**Supplementary Table 2:** [**https://drive.proton.me/urls/FGZ8VBV624#aMOpxj88bEqj**](https://drive.proton.me/urls/FGZ8VBV624#aMOpxj88bEqj)

**Note -** To build the road length data (Supplementary Table 2) the following road types were not considered: "Alley, Pedestrian, Stair, and Trail."

**Supplementary Table 3. Regression analysis.**

After accounting for road density, population density, and testing intensity, the intercept

for Poti and Rustavi was 0.11677, compared to just 0.00079 for all other municipalities. This

difference represents a 148-fold higher baseline disease burden that is independent of the

measured covariates. In contrast to the large difference in baseline risk, the effects of the

individual continuous predictors were identical in both municipality groups. Therefore, road

density, population density and testing emphasis did not act as effect modifiers. Yet, the

relevance of connectivity as a predictor of disease dispersal (as estimated by TP/ km²) was

supported: the coefficient of road density (β = 0.005848) was +100 times higher than those of the

remaining predictors.

| **Regression equation** | |
| --- | --- |
| **Municipality** | |
| Poti & Rustavi (TP%/km²)= | 0.11677 + 0.005848 (RD) - 0.00000196 (PD) + 0.0000447 (TE) |
| Other (TP%/km²)= | 0.00079 + 0.005848 (RD) - 0.00000196 (PD) + 0.0000447 (TE) |

| **Coefficients** | | | | | |
| --- | --- | --- | --- | --- | --- |
| **Term** | **Coefficient** | **SE** | **T value** | ***p* value** | **VIF** |
| **Constant** | 0.00079 | 0.00120 | 0.65 | 0.513 |  |
| **RD** | 0.005848 | 0.000949 | 6.16 | 0.000 | 5.22 |
| **PD** | -0.000002 | 0.000002 | -1.11 | 0.266 | 5.16 |
| **TE** | 0.000045 | 0.000013 | 3.57 | 0.000 | 1.04 |
| **Municipality** |  | | | | |
| Poti & Rustavi | 0.11599 | 0.00217 | 53.54 | 0.000 | 1.53 |
| Other |  | | | | |

**TP**/**km²**: Test positivity % adjusted to municipality area (km²), **RD:** road density (km/km²), **PD**: population density (inhabitants/km²), **TE**: testing emphasis (tests/1000 inhabitants).

**Supplementary Figure 1. Correlation analysis of continuous predictors.** The correlation between road density and population density revealed a positive coefficient, which appeared to be statistically significant (*r*= .89, C.I. 0.88, 0.90, **A**). The remaining correlations were negligible (road density & testing emphasis: *r*= -0.12, CI. -0.16, -0.08, **B**; population density & testing emphasis: *r*= -.04, CI: -0.08, 0.00, **C**). Because none of the assessments revealed a linear relationship, none of these variables significantly related to one another. This means that these three continuous variables are covariates but not effect modifiers of the regression reported in Supplementary Table 3.

**
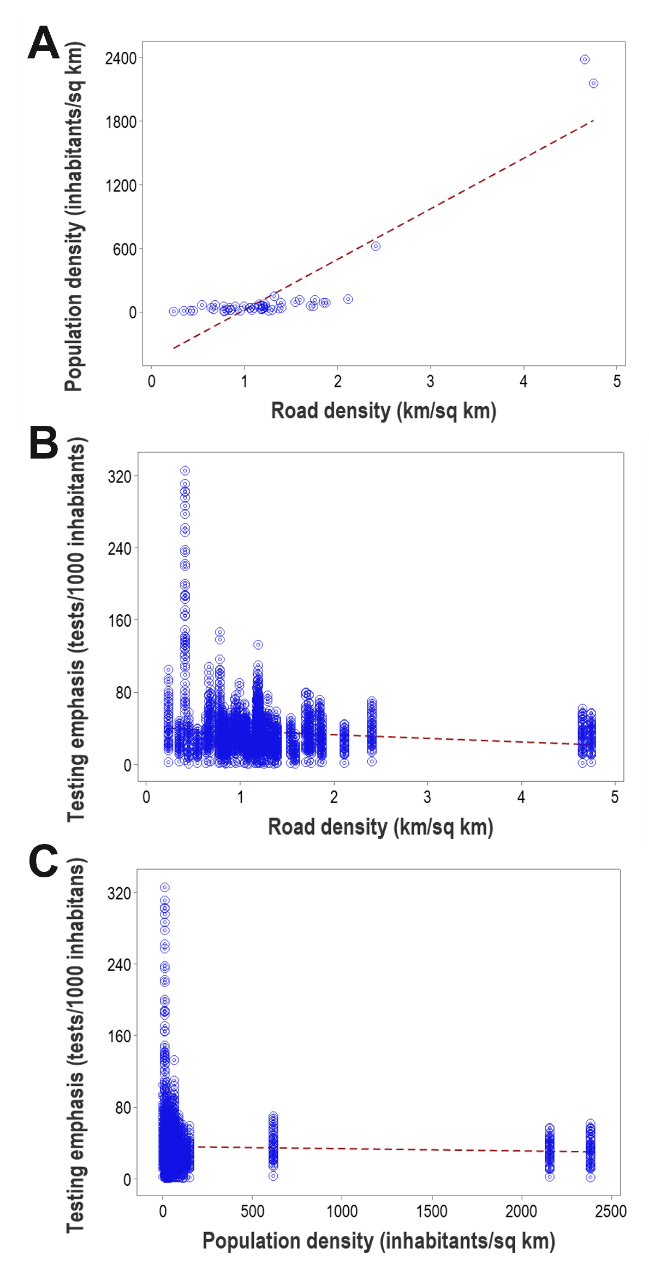
**

**TP**/**km²**: Test positivity % adjusted to municipality area (km²), **RD:** road density (km/km²), **PD**: population density (inhabitants/km²), **TE**: testing emphasis (tests/1000 inhabitants).
